# Familial Pancreatic Cancer Associated with a Shared *BRIP1* Variant: Implications for Homologous Recombination Deficiency and Targeted Therapy

**DOI:** 10.3390/genes17101201

**Published:** 2026-09-29

**Authors:** Nils Degrauwe, Giovanni Dei Tos, Mounir Trimech, Krisztian Homicsko, Antonia Digklia

**Affiliations:** 1 Medical Oncology Service, Hôpital du Valais, 1951 Sion, Switzerland; nils.degrauwe@hopitalvs.ch; 2 Department of Oncology, Lausanne University Hospital and University of Lausanne, 1011 Lausanne, Switzerland; 3 Department of Laboratory Medicine and Pathology, Institute of Pathology, Centre Hospitalier Universitaire Vaudois, Lausanne University, 1011 Lausanne, Switzerland

**Keywords:** pancreatic ductal adenocarcinoma, *BRIP1*, hereditary pancreatic cancer, homologous recombination deficiency, precision medicine

## Abstract

Pancreatic ductal adenocarcinoma (PDAC) remains one of the most lethal malignancies, with a subset occurring in the context of hereditary cancer predisposition. *BRIP1* is involved in homologous recombination repair, and pathogenic germline variants are established risk factors for ovarian cancer. However, its role in pancreatic cancer susceptibility and treatment response remains poorly defined. We report PDAC in a father and son whose tumors independently harbored the same *BRIP1* p.K159E variant of uncertain significance (VUS), with clinical features suggestive of potential homologous recombination deficiency (HRD). A father and son developed PDAC at 70 and 39 years, respectively. Tumor profiling identified the same *BRIP1* p.K159E VUS in both, alongside canonical PDAC-associated alterations. Germline testing was not performed. Both patients showed clinically meaningful sensitivity to platinum-based chemotherapy. The father achieved a partial response to FOLFIRINOX for early metastatic recurrence but subsequently deteriorated and died within one year of diagnosis. The son achieved prolonged disease control with platinum-based chemotherapy and multimodal treatment, including surgery and local therapies for oligoprogression, surviving approximately five years. Maintenance olaparib was briefly administered but discontinued because of hematological toxicity and subsequent disease progression. The occurrence of PDAC in two first-degree relatives, including early-onset disease in the son, together with the same *BRIP1* p.K159E VUS in both tumors, represents an unusual familial and molecular observation. Although germline status and pathogenicity cannot be established, the shared alteration and platinum sensitivity raise the hypothesis that this *BRIP1* variant may warrant further investigation in the context of homologous recombination repair. However, platinum sensitivity alone cannot be considered evidence of homologous recombination deficiency. The uncertain benefit from PARP inhibition further highlights the limitations of inferring therapeutic actionability from an individual HRR gene VUS. Further germline and functional investigation of *BRIP1* alterations in PDAC is warranted.

## 1. Introduction

Pancreatic ductal adenocarcinoma (PDAC) continues to be one of the most devastating malignancies, with a dismal 5-year survival rate of less than 10% [1]. While the majority of PDAC cases are sporadic, approximately 5–10% are thought to have a hereditary basis, often associated with germline mutations in genes involved in DNA repair and cell cycle regulation [2]. One such gene gaining attention in the context of hereditary cancers is *BRIP1* (BRCA1-interacting protein 1), also known as *FANCJ* or *BACH1* [3]. *BRIP1* encodes a DNA helicase that plays a central role in the homologous recombination (HR) pathway of DNA repair by interacting directly with BRCA1 [4]. Germline mutations in *BRIP1* have been well documented in association with hereditary breast and ovarian cancers, conferring a moderate increase in risk [5,6,7]. More recently, the oncologic community has begun to recognize its possible involvement in other malignancies, including colorectal and pancreatic cancers [8]. Response to PARP inhibitors in the context of *BRIP-1* mutated endometrial cancer has been described [9]. Here, we report PDAC in a father and son whose tumors harbored the same *BRIP1* p.Lys159Glu variant of uncertain significance. Although this finding is of interest in the context of familial PDAC, its significance remains unclear, particularly in the absence of germline testing and functional characterization of the variant. This unusual familial observation raises questions regarding the potential relevance of *BRIP1* alterations in pancreatic cancer and their possible implications for homologous recombination repair.

### 1.1. BRIP1: Molecular Function and Cancer Association

*BRIP1* encodes a 1249–amino acid DNA helicase that plays a central role in maintaining genomic integrity. It is recruited to sites of DNA double-strand breaks through direct binding to the tandem BRCT domains of BRCA1 [4]. Structural studies have shown that these BRCT repeats act as phosphoserine/threonine-binding modules, mediating phospho-dependent recognition of repair factors such as *BRIP1*/BACH1 [10]. This interaction is crucial for the proper execution of homologous recombination–mediated repair, and disruption of BRCA1–*BRIP1* binding abrogates checkpoint control and DNA repair efficiency, highlighting a tumor suppressor role for *BRIP1* [4].

The physiological significance of *BRIP1* in genome maintenance is also underscored by the observation that biallelic germline mutations cause Fanconi anemia, a disorder characterized by chromosomal instability, congenital abnormalities, and cancer predisposition [11].

Monoallelic truncating mutations in *BRIP1* have been implicated in hereditary cancer susceptibility, though their impact differs between breast and ovarian cancer [6,7]. In a landmark study of Icelandic women, Rafnar et al. (2011) identified a rare *BRIP1* frameshift mutation (c.2040_2041insTT) that increased ovarian cancer risk more than eightfold [5]. The study also demonstrated that deleterious *BRIP1* variants can reduce lifespan and predispose carriers to multiple cancers. Indeed, two additional cancer types showed nominally significant association: pancreatic cancer (OR = 2.71) and rectal cancer (OR = 2.25).

In addition to the study by Rafnar and colleagues, which reported an association between *BRIP1* mutations and pancreatic cancer risk, other investigations using next-generation sequencing of germline and somatic DNA from pancreatic cancer patients have identified frequent alterations—affecting nearly 10% of cases—in genes involved in double-strand DNA damage repair [5,12]. Among 289 patients affected by PDAC, 28 (9.7%) were found to carry pathogenic or likely pathogenic germline variants in double-strand DNA damage repair genes including 3 (1%) patients with *BRIP1* alterations. All three patients had either personal or familial history of cancer (mainly breast cancer). In addition, one patient had a brother who was also diagnosed with pancreatic cancer [12]. Another large study evaluating prevalence of homologous recombination-related gene mutations across multiple cancer types found homologous recombination deficiency and *BRIP1* alterations in respectively 15.4% and 0.48% of pancreatic cancer patients [13], while only one *BRIP1* mutation was detected in the TCGA pancreatic cancer study [14,15].

### 1.2. Potential Implications for Treatment

Homologous recombination deficiency (HRD) plays an important role in tailoring treatment for pancreatic ductal adenocarcinoma (PDAC). The best-characterized genes are *BRCA1*, *BRCA2*, and *PALB2*, where evidence supports the use of platinum-based chemotherapy. Indeed, germline mutated patients show high overall response rates of 58% compared to 21% in wild type matched control group [16]. Furthermore, patients with borderline resectable PDAC harbouring germline *BRCA1/2* mutations were more likely to achieve pathologic complete response (44.4% vs 10%) compared to BRCA non-carriers [17] under FOLFIRINOX treatment.

By contrast, impact on therapeutic decision regarding chemotherapy choice for other HRD-associated genes—such as *ATM*, *CHEK2*, *RAD51*—are less well defined and limited to case reports [18,19]. Regarding *BRIP1*, the case report mentioned above, describes remarkable responses to platinum-based chemotherapy (mFOLFIRINOX) with normalization of tumor marker CA19-9 [20].

Beyond chemotherapy, PARP inhibitors have emerged as an important therapeutic option for a broad range of solid tumors harboring homologous recombination deficiency gene alterations, particularly *BRCA1/2*. In pancreatic cancer, the strongest evidence comes from the POLO trial, in which patients with germline *BRCA1/2* mutations were randomized to receive olaparib as maintenance therapy, provided they had no disease progression during first-line platinum-based chemotherapy. While an overall survival (OS) benefit was not demonstrated, a significant improvement in progression-free survival (PFS) was observed (7.4 months vs. 3.8 months in the placebo group) [21]. In contrast, for other HRD-associated genes such as *ATM*, *CHEK2*, and *RAD51*, PARP inhibitor activity is less well defined, with supporting evidence largely restricted to case series [22,23]. A clinical case was published in 2022 documenting a woman with *BRIP1*-mutated pancreatic adenocarcinoma who developed leptomeningeal disease (LMD), a rare and aggressive form of metastasis in PDAC [20]. The patient initially responded well to modified FOLFIRINOX, followed by maintenance therapy with olaparib, a PARP inhibitor. However, she experienced progression within 9 months, including brain metastases and extensive LMD leading to death within 11 months of diagnosis.

### 1.3. Clinical Case: Familial Occurrence of Pancreatic Ductal Adenocarcinoma (PDAC) in a Father and Son with an Identical BRIP1 p.Lys159Glu Variant

The father

Mr. X is a 70-year-old gentleman who presented to the emergency room with upper abdominal pain, weight loss, and pale stools. Laboratory tests revealed elevated lipase levels (964 U/L) and hyperbilirubinemia (64 µmol/L), along with a mixed pattern of liver function test abnormalities. Baseline characteristics of the patients are summarized in Table 1.

CT scan revealed a suspicious lesion in the uncinate process of the pancreas, measuring 3.4 cm, causing obstruction of the bile duct and the main pancreatic duct, with associated pancreatitis. Endoscopic ultrasound-guided biopsy confirmed a moderately differentiated adenocarcinoma. A biliary stent was placed. Baseline CA 19-9 was 11,328 kU/L. Following multidisciplinary team (MDT) discussion and review, which ruled out metastatic disease, the patient was referred for surgery and underwent a cephalic duodenopancreatectomy (Whipple procedure). Pathology confirmed a grade 2 pancreatic ductal adenocarcinoma, classified as pT2 (3.4 cm), pN2 (6/20), M0, L1, V1, Pn1, R0. Two months after surgery, follow-up CT showed progression of hepatic lesions and multiple bilateral pulmonary nodules, consistent with metastatic disease.

**Figure 1:**
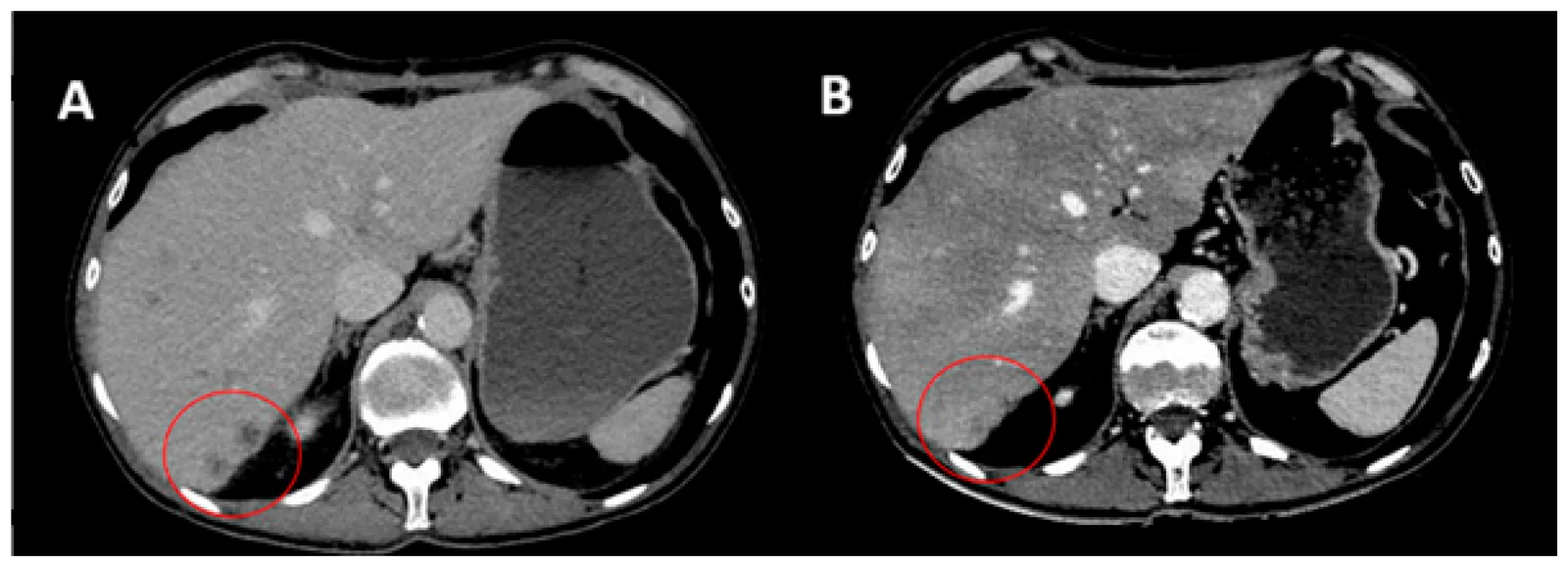
“Father” Baseline (**A**) CT scan after 3 months (**B**) of palliative FOLFIRINOX chemotherapy.

Over the course of 6 months, the patient received first-line palliative chemotherapy with FOLFIRINOX, later dose-reduced due to thrombocytopenia. The first restaging CT scan, performed after 3 months of chemotherapy, revealed evidence of a partial radiological response (Figure 1).

Molecular analyses were performed on tumor tissue from the pancreatectomy specimen using next-generation sequencing (NGS) with an in-house-developed panel covering the full-coding sequences of 423 cancer-associated genes. The analysis identified variants in *BRIP1*, *TP53*, *KRAS*, and *RB1* (Table 2), as well as amplifications of *CCNE1*, *AKT2*, and *LTBP4*. Biomarker analysis was negative for HER2, PD-L1, and pan-TRK, and the tumor mutational burden was low. The patient was discussed at our molecular tumor board. The *BRIP1* p.Lys159Glu variant was further evaluated, in silico structural analysis suggested that Lys159 is located within a positively charged region that may function as a nuclear localization signal. Replacing lysine with the negatively charged glutamic acid could therefore affect *BRIP1* nuclear localization. However, this remains a computational prediction and has not been confirmed experimentally. To the best of our knowledge, to date, there is no functional evidence showing that the p.Lys159Glu variant impairs *BRIP1* nuclear localization or function. Given the role of *BRIP1* in homologous recombination repair, the use of olaparib was proposed as a later-line treatment, acknowledging that supporting evidence is limited.

However, the patient developed severe hyperbilirubinemia and cholestasis, preventing initiation of next-line therapy. Imaging performed shortly after the 6-month FOLFIRINOX treatment demonstrated progression of hepatic, peritoneal, and nodal metastases. At that stage, given clear oncological progression, patient preference and limited treatment options, the recommendation was best supportive care with palliative management focused on comfort and pain control.

The son

The patient aged 39 with no significant medical history, has experienced heartburn and epigastric pain radiating to the periumbilical area, initially treated with antacids. His condition worsened with the onset of jaundice and weight loss of 4 kg. An abdominal CT scan showed biliary dilation caused by probable pancreatic head mass. ERCP and EUS revealed diffuse infiltration of the pancreatic head and a hepatic hilum lymph node, intra-ductal infiltration of the common bile duct, and signs of pancreatitis, leading to the placement of two stents. Biopsy confirmed infiltrating adenocarcinoma of the pancreas.

**Figure 2:**
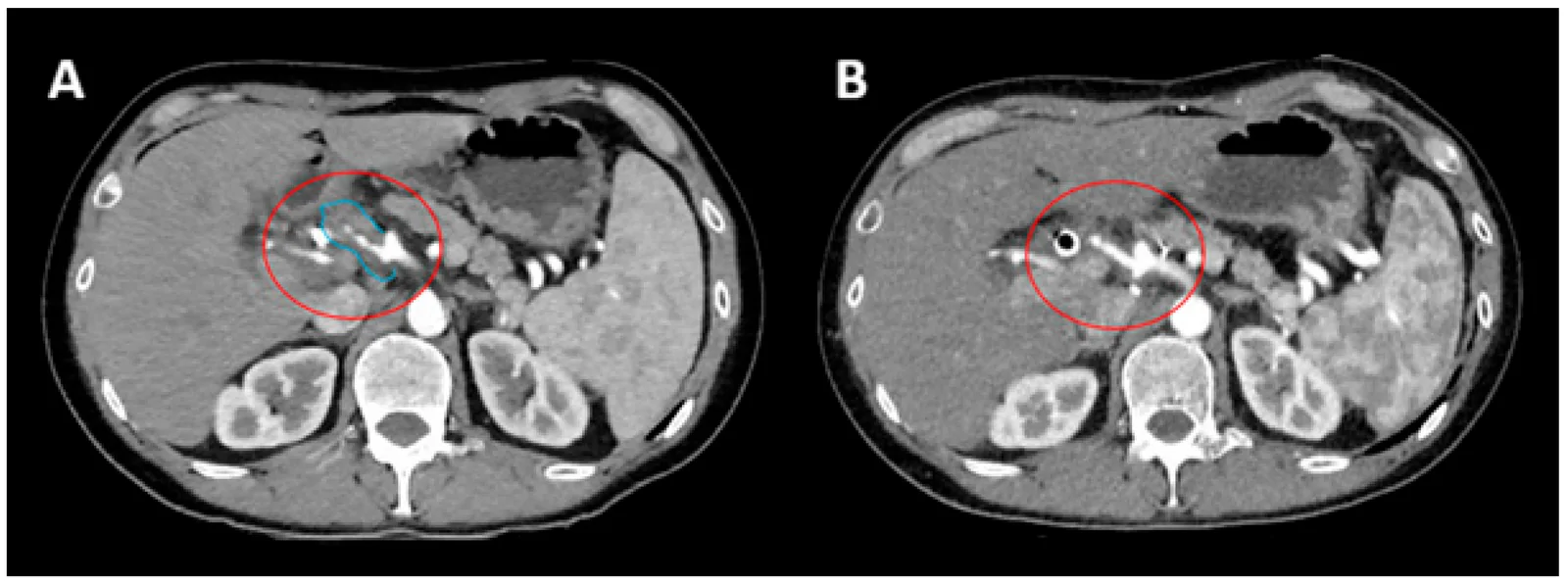
Baseline (**A**) CT scan demonstrating vascular involvement (around hepatic artery in blue) and partial response (**B**) after FOLFIRINOX based chemotherapy.

Baseline thoraco-abdominal CT demonstrated peri-pancreatic infiltration involving the gastroduodenal artery and contacting the hepatic artery, main portal vein stenosis without thrombus (Figure 2). MDT recommended induction chemotherapy. The patient received twelve cycles of neoadjuvant FOLFIRINOX. Restaging with CT showed disease stability with a 6% reduction per RECIST 1.1, but the tumor remained inoperable. The patient received 3 additional months of FOLFIRI chemotherapy, achieving a partial response (Figure 2). Following multidisciplinary discussion, a Whipple procedure was recommended following resolution of arterial encasement. The patient subsequently underwent surgery. Histopathological examination demonstrated a ypT2 tumor with ypN2 nodal involvement (4 of 12 nodes positive), lymphatic and vascular invasion (L1, V1), perineural invasion (Pn1), moderate differentiation (G2), and negative resection margins (R0).

The patient was enrolled in a clinical trial with adjuvant chemotherapy, nivolumab, and an experimental vaccine from 3 months after surgery, with no recurrence until local progression at the pancreatic plexus confirmed by EUS biopsy 2 years after surgery.

At that point first-line palliative chemotherapy with gemcitabine and nab-paclitaxel was administered. CT after 9 months of chemotherapy showed significant growth of three bilateral lower lobe lung nodules, and radiotherapy (54 Gy in 3 fractions) was delivered to a progressive right lower lobe lesion. PET-CT performed 16 months after start of chemotherapy showed increased metabolism at the hepatic hilum and progression of known lung nodules without new lesions. Tumor board recommended local radiotherapy to the primary site and cryoablation for lung lesions. The patient received chemo-radiotherapy with weekly carboplatin (AUC2), stopped after two cycles due to thrombocytopenia, alongside peri-hilar liver radiotherapy (54 Gy in 27 fractions).Shortly after completion of chemo-radiotherapy CT showed progression with peritoneal carcinomatosis, leading to cancellation of lung-directed treatments. Chemotherapy with reduced-dose gemcitabine (75%) and nab-paclitaxel (60%) was continued.

Molecular analyses (Table 3) revealed several variants, including *KRAS*, *TP53*, and *SMAD4* mutations, as well as a *BRIP1* p.Lys159Glu variant, which was also present in the patient’s father. This variant was detected at approximately 50% VAF in both tumors, despite their low tumor cellularity (20–30%), suggesting a constitutional heterozygous variant rather than a tumor specific somatic event. No additional somatic *BRIP1* variant or clear evidence of *BRIP1* loss was identified in the NGS data from either tumor. However, given the low tumor cellularity, the sensitivity of NGS based copy-number and loss of heterozygosity (LOH) analyses is limited, and the presence of a somatic second hit or *BRIP1* loss cannot be reliably excluded. The *BRIP1* p.Lys159Glu variant is classified as a variant of uncertain significance (VUS) in available clinical variant databases. It is not reported in population databases, and there is currently insufficient clinical or functional evidence to support a pathogenic role. HER2 immunohistochemistry showed a score of 2+, whereas FISH was negative. Molecular tumor board suggested PARP inhibitors or trastuzumab deruxtecan as later-line options.

24 months after start of gemcitabine and nab-paclitaxel, maintenance with gemcitabine plus olaparib was initiated based on the potential functional involvement of *BRIP1* in homologous recombination repair but complicated by thrombocytopenia requiring dose reductions; olaparib was stopped after ~2 months and 2 months later gemcitabine as well. Given the short treatment exposure and dose modifications, no conclusion regarding sensitivity or resistance to PARP inhibition could be drawn.

Restaging CT showed progression of peritoneal disease. Tumor board recommended palliative radiotherapy, portal stent revision, and switch to trastuzumab deruxtecan.

The subsequent course was complicated by recurrent infections (cholangitis with bacteremia and port-a-cath infections), requiring repeated hospitalizations and limiting further systemic therapy. The patient died 5 years after initial diagnosis.

## 2. Conclusions

We report PDAC in a father and son whose tumors both carried the same *BRIP1* p.Lys159Glu variant of uncertain significance. The presence of PDAC in two first-degree relatives, including early-onset disease in the son, is notable. However, germline testing was not performed, and the functional significance of the variant is unknown. These cases therefore do not establish hereditary transmission or a causal role for *BRIP1* in pancreatic cancer predisposition. Given the allelic frequency suggestive of a germline origin, formal germline testing was requested. However, this request was declined by the genetics team. The variant is currently classified as a variant of uncertain significance (VUS), and although *BRIP1* is implicated in homologous recombination repair, its established association is primarily with ovarian cancer susceptibility. The genetics team concluded that, even if the variant were confirmed to be germline, its VUS classification would not have immediate clinical utility for patient management or familial counseling. Therefore, germline testing was not pursued. Without germline testing or functional assessment of the variant, its hereditary nature and potential contribution to pancreatic cancer susceptibility remain uncertain. This case therefore raises the possibility of an association but does not establish causality, and further investigation of *BRIP1* variants in familial PDAC is needed. Both tumors also harbored KRAS codon 12 mutations, although the variants differed (G12D in the father and G12V in the son). KRAS mutations are found in approximately 90% of PDACs and are considered an early event in pancreatic carcinogenesis. G12D and G12V are among the most commonly observed variants [24]. Their presence in both tumors is therefore not unexpected and, importantly, the different substitutions do not suggest a shared familial alteration. From a therapeutic perspective, KRAS remains an area of active investigation, with several mutant-specific and broader RAS-targeted approaches currently in clinical development and entering practice.

Both patients had clinically meaningful responses to platinum-based chemotherapy. Although platinum sensitivity can occur in PDAC with HRD, particularly in tumors with pathogenic BRCA1/2 or PALB2 alterations, it is not specific to HRD and does not establish an HR-deficient phenotype. Because the *BRIP1* variant was not functionally characterized and HRD was not assessed genomically, these treatment responses should be interpreted as clinical observations rather than evidence of *BRIP1*-related HRD. While the father’s disease followed an aggressive course, with survival of less than one year, the son has exhibited a more indolent trajectory and lived more than five years after diagnosis. It remains unclear whether this difference reflects inherent biological heterogeneity between their tumors or is influenced by the son’s exposure to personalized adjuvant vaccine and immunotherapy. The son received olaparib for only a short period, with treatment limited by hematological toxicity. This exposure was insufficient to assess sensitivity or resistance to PARP inhibition and should not be interpreted as evidence for or against the therapeutic relevance of the *BRIP1* variant.

Continued reporting of *BRIP1* (and other HRR genes)-related pancreatic cancer cases, along with mechanistic studies and clinical trials, will be essential to clarify the mutation’s penetrance, therapeutic relevance, and value in cancer prevention strategies.

## Figures and Tables

**Table 1:** Baseline patients characteristics.

Parameter	Ref Range	Father	Son
WBCs	4–10 G/L	9.2	6.3
RBCs	4.4–5.8 T/L	3.65	4.48
Hb	133–177 g/L	116	144
PLT	150–350 G/L	299	192
Creatinine	62–106 µmol/L	72	66
eGFR	mL/min/1.73 m^2^	>90	>90
ASAT	14–50 U/L	199	307
ALAT	11–60 U/L	280	667
Alkaline phosphatase	36–108 U/L	382	546
Gamma-GT	10–71 U/L	450	1625
Total bilirubin	0–21 µmol/L	64	79
Direct bilirubin	0–10 µmol/L	56	43
Lipase	13–60 U/L	964	41
Ca 19-9	<37 kU/L	11,328	39
Alcohol use		No	No
Smoking history		4 pack years, stopped 2 years before cancer diagnosis	8.5 pack years, stopped at cancer diagnosis
Medical history		Type II diabetes	Nihil

**Table 2:** Variants identified by next-generation sequencing in the pancreatic adenocarcinoma of the father.

Gene	RefSeq ID	Variant	VAF	Coverage	ACGS Class
*BRIP1*	NM_032043.3	c.475A>G (p.Lys159Glu)	48%	460X	Uncertain significance
*TP53*	NM_000546.5	c.672G>A (p.Glu224Glu)	23%	2091X	Pathogenic
*KRAS*	NM_004985.5	c.35G>A (p.Gly12Asp)	16%	591X	Pathogenic
*RB1*	NM_000321.2	c.2106+3_2106+23del (splice-site)	11%	201X	Uncertain significance

The estimated tumor content was 30%, and the mean sequencing coverage was 1223X. Variant allele frequencies (VAFs) and sequencing coverage are indicated for each variant. Variants were classified according to the Association of Clinical Genomic Science (ACGS) guidelines: class 3, variant of uncertain significance; class 4, likely pathogenic; and class 5, pathogenic.

**Table 3:** Variants identified by next-generation sequencing in the pancreatic adenocarcinoma of the son.

Gene	RefSeq ID	Variant	VAF	Coverage	ACGS Class
*BRIP1*	NM_032043.3	c.475A>G (p.Lys159Glu)	49%	508X	Uncertain significance
*TP53*	NM_000546.5	c.818G>A (p.Arg273His)	9%	1306X	Pathogenic
*KRAS*	NM_004985.5	c.35G>T (p.Gly12Val)	10%	1166X	Pathogenic
*SMAD4*	NM_005359.6	c.1598T>C (p.Leu533Pro)	9%	1074X	Likely pathogenic
*EPHB1*	NM_004441.5	c.475A>G (p.Lys159Glu)	11%	508X	Uncertain significance
*TERT*	NM_198253.3	c.3032+7C>T (splice-site)	10%	1115X	Uncertain significance
*TSHR*	NM_000369.3	c.929G>A (p.Arg310His)	9%	1025X	Uncertain significance
*AURKA*	NM_198437.3	c.1154C>A (p.Ala385Glu)	9%	1209X	Uncertain significance

The estimated tumor content was 20%, and the mean sequencing coverage was 943X. Variant allele frequencies (VAFs) and sequencing coverage are indicated for each variant. Variants were classified according to the Association of Clinical Genomic Science (ACGS) guidelines: class 3, variant of uncertain significance; class 4, likely pathogenic; and class 5, pathogenic.

## Data Availability

The data supporting the findings of this report are not publicly available due to patient privacy and confidentiality considerations. Additional information may be available from the corresponding author upon reasonable request, subject to applicable ethical and legal restrictions.

## Abbreviations

*BRIP1*: BRCA1-interacting protein 1; CT, computed tomography; ERCP, endoscopic retrograde cholangiopancreatography; EUS, endoscopic ultrasound; FOLFIRI, folinic acid, fluorouracil, and irinotecan; FOLFIRINOX, folinic acid, fluorouracil, irinotecan, and oxaliplatin; HER2, human epidermal growth factor receptor 2; HR, homologous recombination; HRD, homologous recombination deficiency; MDT, multidisciplinary team; NGS, next-generation sequencing; PARP, poly(ADP-ribose) polymerase; PDAC, pancreatic ductal adenocarcinoma; PD-L1, programmed death-ligand 1; RECIST, Response Evaluation Criteria in Solid Tumors; TMB, tumor mutational burden; VAF, variant allele frequency; VUS, variant of uncertain significance.
